# Computing Power and Sample Size for Case-Control Association Studies with Copy Number Polymorphism: Application of Mixture-Based Likelihood Ratio Test

**DOI:** 10.1371/journal.pone.0003475

**Published:** 2008-10-22

**Authors:** Wonkuk Kim, Derek Gordon, Jonathan Sebat, Kenny Q. Ye, Stephen J. Finch

**Affiliations:** 1 Department of Mathematics and Statistics, University of South Florida, Tampa, Florida, United States of America; 2 Department of Genetics, Rutgers University, Piscataway, New Jersey, United States of America; 3 Cold Spring Harbor Laboratory, Cold Spring Harbor, New York, United States of America; 4 Department of Epidemiology and Population Health, Albert Einstein College of Medicine, Bronx, New York, United States of America; 5 Department of Applied Mathematics and Statistics, Stony Brook University, Stony Brook, New York, United States of America; Vrije Universiteit Medical Centre, Netherlands

## Abstract

Recent studies suggest that copy number polymorphisms (CNPs) may play an important role in disease susceptibility and onset. Currently, the detection of CNPs mainly depends on microarray technology. For case-control studies, conventionally, subjects are assigned to a specific CNP category based on the continuous quantitative measure produced by microarray experiments, and cases and controls are then compared using a chi-square test of independence. The purpose of this work is to specify the likelihood ratio test statistic (*LRTS*) for case-control sampling design based on the underlying continuous quantitative measurement, and to assess its power and relative efficiency (as compared to the chi-square test of independence on CNP counts). The sample size and power formulas of both methods are given. For the latter, the CNPs are classified using the Bayesian classification rule. The *LRTS* is more powerful than this chi-square test for the alternatives considered, especially alternatives in which the at-risk CNP categories have low frequencies. An example of the application of the *LRTS* is given for a comparison of CNP distributions in individuals of Caucasian or Taiwanese ethnicity, where the *LRTS* appears to be more powerful than the chi-square test, possibly due to misclassification of the most common CNP category into a less common category.

## Introduction

Large-scale copy number polymorphisms (CNPs) are a recently discovered feature of human genomic architecture [1]. As reported by Sebat et al. [1], large-scale copy number polymorphisms (CNPs) (about 100 kilobases and greater) contribute substantially to genomic variation among normal humans. These authors documented CNPs of 70 different genes within CNP intervals, including genes involved in neurological function, regulation of cell growth, regulation of metabolism, and several genes known to be associated with disease. For example, investigators have documented that copy number variation of the region encompassing the *CCL3L1* gene [MIM 601395] is associated with HIV/AIDS susceptibility [2] [MIM 609423]. Other investigators have documented that copy number variation of the orthologous rat and human *FCGR3* genes [MIM 146740] is a determinant of susceptibility to immunologically mediated glomerulonephritis [3], [4] [MIM 610665]. Additional recent publications suggest that CNPs may play a role in cardiovascular disease [5], lipoprotein and metabolic phenotypes [6], nervous system disorders [7], age-related macular degeneration [8], [9] [MIM 610149], autism [10], cancer [1], [11], and schizophrenia [12]. More generally, CNPs may play an important role in disease etiology for common, complex traits. Additionally, CNPs, like SNPs and microsatellite markers, may have different distributions for populations with different ethnicities [13], [14].

Case-control genetic association designs can be a powerful way to map disease susceptibilty genes, particularly for diseases with smaller effect sizes [15], [16], [17], [18], [19]. In such designs, unrelated cases (with the phenotype of interest) and controls (who do not have the phenotype) are genotyped usually for thousands to hundreds of thousands of single nucleotide polymophisms (SNPs) across the human genome. Standard statistical analyses include the chi-square test of independence or the linear trend test [20], [21] applied to the individual SNP genotype counts from cases and controls. These genotypes are usually determined through use of clustering algorithms applied to underlying quantitative measurements (e.g., see [22]).

Compared to SNP genotyping technologies, procedures for calling CNPs are less developed and less accurate [23]. Earlier CNP studies focused on discovery [24], with copy number changes being called using data from a single array, comparing DNA from an individual with a reference DNA sample. Recently developed methods classify known CNPs using array data collected from a large group of individuals. One method classifies known CNPs from the distribution of a univariate quantitative measure (C. Yoon; manuscript in preparation). Such a quantitative measure is either an average log fluorescent intensity ratio (between sample and reference DNA) over multiple probes representing the CNP, or the log-intensity ratio of the best probe within the CNP region.

One reason for the relative difficulty of CNP classification is that such classification is determined by relative intensity of a signal at a probe (or probe sets). In contrast, the two alleles of a SNP have two distinct nucleotides that can be represented by two distinct probes. Moreover, for multi-allelic CNPs, only the total number of copies (or categories) and not the alleles are observed for each individual. As an example, for a CNP locus of three alleles, with 1 copy, 2 copies and 3 copies respectively and probe intensity proportional to the number of copies, an intensity observation of 4 can be a genotype of 2/2 copies or a genotype of 1/3 copies.

**Figure 1:**
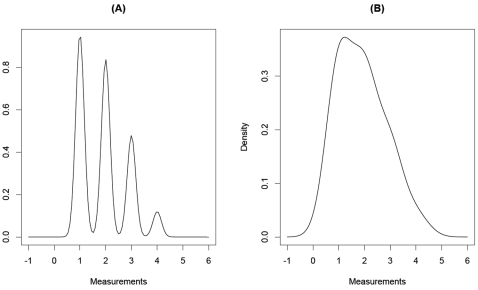
1a and 1b. In this figures, we present probability density plots for statistical distributions that are mixtures of four univariate normal distributions with equally spaced means 1, 2, 3, and 4, and a common variance. In Figure 1a, the variance of each component distribution is 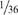. In Figure 1b, the variance of each component distribution is ¼.

Consider the pictorial examples in Figures 1a and 1b, which are created to represent a hypothetical CNP with a total of four different copy number categories (labeled “1” through “4”). For each category, different subjects will have a quantitative measure following a fixed continuous distribution, whether or not the subject is a case or control. The case category frequencies are then the mixing proportions of the component distributions [25]; similarly for the controls. In Figure 1a, the quantitative measures for CNP category *i*, 1≤*i*≤4 comes from a univariate normal distribution with mean *i* and variance 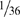 each. Studying the figure, we see there is clear separation among the component normal distributions, so that classification of individuals into categories 1,…, 4 is highly accurate [26]. An example where classification is more problematic is presented in Figure 1b. In this figure, the CNP quantitative measures for subjects have normal distributions with the same means as in Figure 1a, but with variance ¼ each. That is, for each univariate distribution in Figure 1b, the variance is nine times that of the variance in Figure 1a, resulting in greater overlap among component distributions. As suggested by Figure 1b, when the component distributions have more overlap, the rate of misclassifying an individual having true CNP category *i* as having CNP category *j*≠*i* is much higher. It has been reported that the chi-square test of independence loses power as the misclassification rate increases [26], [27], [28].

An additional concern is that the CNP category that increases risk may occur with low frequency, as is often the situation with Mendelian diseases [29]. In case-control association studies using SNPs with low at-risk allele frequency, an increase in the genotype misclassification error rates requires indefinitely large increases in sample size to maintain constant power [30], [31]. We hypothesize that CNP classification errors may lead to underpowered studies when the at-risk CNP category has low frequency. Challenges of performing association studies using CNP data were recently documented by McCarroll and Altshuler [32], who note, “To the extent that the precise allelic state of any DNA is not well measured, power declines.” We raise the question: *Is there a more (statistically) powerful method of using CNP data when testing for association with a complex trait than the usual chi-square test of independence?*

To answer this question, we propose use of the likelihood ratio test statistic (*LRTS*) comparing the mixing proportions of cases and controls estimated from the underlying quantitative measures for CNPs. Rather than assign classifications to each individual's CNP, we perform a test of association on the CNP quantitative measure. We present an analytic solution to computation of power and sample size calculations for genetic association with CNP quantitative measures. We then calculate the efficiency of the chi-square test of independence using Bayesian classification compared to the *LRTS* to examine which test statistic has greater power for a wide range of trait specifications. By efficiency, we mean the ratio of sample size requirements for the chi-square test of independence and *LRTS*, respectively, for a fixed power and type I error rate. Finally, we demonstrate the use of the *LRTS* for differences in the mixing proportions of the CNP categories between two ethnic groups for a CNP with relevance to a genetic disease.

## Methods

### Notation

The following notation is used throughout this work:

*X_α_* = A continuous random variable representing the CNP quantitative measure; *α* is an index indicating control (*α* = 1) or case (*α* = 2) status.

The number of controls is *n*_1_, and cases *n*_2_, with *N* = *n*_1_+*n*_2_ and 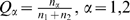, which is the proportion of controls or cases in the total sample.

*d* = The number of CNP categories; the subscript *i* indexes the category, 1≤*i*≤*d*.

*f*(*x*|*θ_i_*,*η*) = The probability density function (pdf) of the continuous random variable *X* = *x*, conditional on the CNP category. This pdf is a function of the parameters *θ_i_* and *η*, where *η* is a parameter that is constant for all component distributions. For example, if *f* is a normal pdf, then *θ* *_i_* is the mean and *η* is the variance.


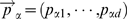
 = A vector of mixing proportions; here, the values *p_αi_*, 1≤*i*≤*d*, are the proportions of CNP category *i* in the *α* affection status class (*α* = 1 for controls, *α* = 2 for cases). Under the null hypothesis, *p*_1*i*_ = *p*_2*i*_.

*p*_0*i*_ = *Q*_1_ *p*_1*i*_+*Q*_2_ *p*_2*i*_. Since, under the null hypothesis, *p*_1*i*_ = *p*_2*i*_, then *p*_1*i*_ = *p*_2*i*_ = *p*_0*i*_, under the null.

*q_i_* = The CNP category frequencies in the population from which cases and controls are drawn.


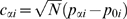
 = A parameter needed for specification of the alternative hypothesis and hence power and sample size calculations.


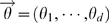
 = A vector of parameters for the probability density functions *f*(*x*|*θ_i_*,*η*). In the examples used here, *θ_i_* is the mean of the CNP category *i* distribution. Also, in the efficiency calculations reported later, *θ_i_*_+1_−*θ_i_* = 1, *i* = 1,…, *d*−1. The separation 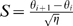 is the number of standard deviations between adjacent CNP category means.

### Probability density function of the CNP quantitative measure

The probability density function of the random variable *X_α_* is given by 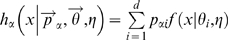, where we assume the number of categories *d* is known and equal in both cases and controls. Given a CNP category *i*, the underlying pdf *f*(·) is the same for cases and controls. When *f*(*x*|*θ_i_*,*η*) is a normal distribution, we specify that the variance (*η*) is equal across all CNP categories *i* and affection statuses *α*. While these specifications are not critical for performing power and sample size calculations, they may be advantageous when performing mixture analyses of real data. For instance, there may be convergence problems for the computed maximum likelihood of a univariate normal mixture if one allows the category variances to be unequal. Methods such as those proposed by Hathaway [33], [34] may be used when the equal variance assumption does not hold.

### Likelihood function

The likelihood function under the null hypothesis is given by:
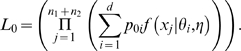
(1)The likelihood function under the alternative hypothesis is given by:
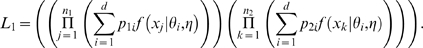
(2)Computationally, *L*_0_ and *L*_1_ are calculated by using the maximum likelihood estimates (MLEs) of the parameters.

### LRTS

In this work, we consider two test statistics: (1) the *LRTS* applied directly to the CNP quantitative measures for cases and controls; and (2) the chi-square test of independence applied to 2×*d* tables after the CNP quantitative measures have been classified into one of *d* categories for cases and controls using a Bayesian classification rule (see section immediately following). The *LRTS* (1) is defined as
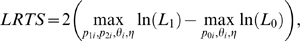
(3)where the likelihoods are defined in equations (1) and (2).

### Bayesian classification rule for univariate CNP quantitative measures

To categorize CNP quantitative measures into a CNP category, we consider a classification formula based on Bayes rule [35]. Since this approach minimizes the expected cost of misclassification, as proven in Anderson [36], it is a well-accepted approach. An observation *x* is assigned to CNP category *i* if and only if 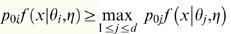, where *p*_0*i*_ = *Q*_1_ *p*_1*i*_+*Q*_2_ *p*_2*i*_, (defined above – Notation). For an example with *d* = 3 copy number categories and a normal CNP category distribution, application of the Bayes rule yields:

*x* is placed in the left-most component if *x*<min(γ_12_,γ_13_),
*x* is placed in the middle component if γ_12_<*x*<γ_23_,
*x* is placed in the right-most component if max(γ_13_,γ_23_)<*x*,

where 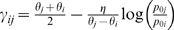, 1≤*i*<*j*≤3. In applications, (*γ*_12_,*γ*_13_,*γ*_23_) are estimated using the MLEs of the parameters.

### Simulation studies to verify asymptotic null distribution of chi-square test with Bayesian classification

We perform simulation studies to verify the accuracy of the asymptotic null distribution of the chi-square test of independence applied to CNP counts after classification using the Bayesian classification rule (described above). We consider two settings each of sample size and mixing proportion vectors (a total of four settings). Our mixture model is a mixture of four univariate normal distributions with consecutive mean distances *θ_i_*_+1_−*θ_i_* = 1 unit apart. Separations are fixed to be 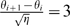. We specify sample sizes *n*_1_( = *n*_2_) = 200 *or* 500, and mixing proportions 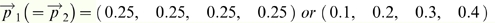.

### Computing asymptotic power for the LRTS of the CNP quantitative measure

The asymptotic distribution of the *LRTS* under the null hypothesis follows a 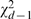 distribution under certain conditions [37] (referred to as “classic regularity conditions”); and the asymptotic power under the alternative specified hypothesis 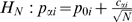 can be calculated using the non-central chi-square distribution 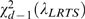 with the non-centrality parameter (NCP) *λ_LRTS_* given in Appendix S1.

### Computing asymptotic power for chi-square test of independence

The 2×*d* test under an alternative hypothesis *H_N_* asymptotically follows a non-central chi-square distribution [38]. When the component distributions have more overlap, the misclassification rates are much higher. If the misclassification error mechanism is random and non-differential, the observed classification probabilities *p* ^*^ can be written in terms of a matrix of classification probabilities *ε* = (*ε_ij_*), where *ε_ij_* = Pr (subject's observed genotype = *i*|subject's true genotype = *j*). The power for the chi-square test of independence with misclassification errors can be calculated from the NCP λ*_CS_* [27], [28], [38], where
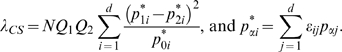



### Genetic model parameters for efficiency analysis

We calculate the efficiency of the chi-square test on 2×*d* contingency tables with respect to the *LRTS* on CNP quantitative measures for two genetic models of inheritance (MOI) associated with CNPs that have been documented as a possible MOI for CNPs [2], [39]. We first specify the disease prevalence *>φ*, the population frequencies *q_i_* for CNP category *i*,1≤*i*≤*d*, and the relative risks *R_i_* of becoming affected, given that an individual has CNP category *i*. We then compute the penetrances *g_i_* = Pr(*affected*|*CNP*_*category* = *i*), where we specify *R_i_* = *g_i_*/*g*_1_, so that the reference *CNP category* relative risk is 1. The reference CNP category may be chosen arbitrarily without loss of generality. The penetrances are given by 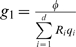, and *g_i_* = *R_i_g*_1_. Using Bayes Theorem, the CNP category mixing proportions conditional on affection status are:
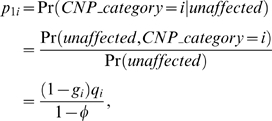
(4)

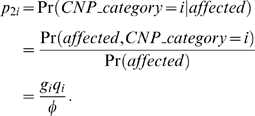
For our comparative analyses, we set *d* = 4, *q*_1_ = 0.4, *q*_2_ = 0.35, *q*3 = 0.2, *q*_4_ = 0.05, and *>φ* = 0.05. In the first (Dosage) model, the risk of becoming affected increases geometrically with increase in CNP category. We specify *R*_2_ = 1.8, *R*_3_ = 1.8^2^ = 3.24, and *R*_4_ = 1.8^3^ = 5.83, so that risk increases by a factor of 1.8 for each increase in CNP category.

In the second (Extremes) model, risk of becoming affected increases for CNP categories 1 and *d* and decreases for all other categories. For this work, we specify *R*_2_ = 0.3, *R*_3_ = 0.3, and *R*_1_ = *R*_4_ = 1. Finally, we set the means to be equally spaced for all components. Specifically, *θ_i_* = *i* for comparative analyses so that separation is given by 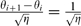.

### Simulation studies to verify asymptotic null and alternative distributions of LRTS

We perform simulation studies to verify the accuracy of the asymptotic null and alternative distributions of the *LRTS*. For the null distribution simulations, we consider two settings each of sample size and mixing proportion vectors (a total of four settings). For the alternative distribution simulations, we consider one setting of sample size and two different MOIs (a total of two settings). Also, for both sets of simulations, our mixture model is a mixture of four univariate normal distributions with consecutive mean distances *θ_i_*_+1_−*θ_i_* = 1 unit apart. Separations are fixed to be 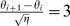.

For the null distribution simulations, we specify sample sizes *n*_1_( = *n*_2_) = 200 *or* 500, and mixing proportions 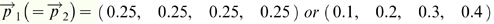. For the alternative distribution simulations, sample sizes are *n*_1_ ( = *n*_2_) = 200, and mixing proportions are determined using equations (4) with the specified parameters (including CNP population frequencies) for the Dosage and Extremes MOIs, given above (Methods - Genetic model parameters for efficiency analysis).

To find the global maximum (equations (1) and (2)), we use Expectation-Maximization algorithms (EM). A small pilot study found that there were typically three relative maxima under the null specification and two under the alternative. Consequently, we use 100 random starting points (RSPs) for parameter estimation under the null distribution simulations and 50 RSPs for the estimation under the alternative distribution simulations. EM algorithm computations are performed using MCLUST in the R programming environment [40]. For each RSP, the convergence tolerance is set at 10^−5^ and the maximum iteration number is set at 300.

### Efficiency of the chi-square test relative to the LRTS

The efficiency of the 2×*d* test relative to the *LRTS* is denoted *Eff* and is the ratio 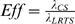 of the NCP of the chi-square test to the NCP of the *LRTS*. When the relative efficiency is less than 1, the chi-square test requires a larger sample size to achieve the same power as the *LRTS*, given that both tests have the same level of significance. For example, if the relative efficiency of the 2×*d* test is 0.8, the 2×*d* test requires 100 observations to have the same power as the *LRTS* using 80 observations.

### Example CNP data for two ancestral populations

Since recent work documents different CNP distributions in different ethnic populations [41], [42] we apply our *LRTS* to test for differences in mixing proportions of CNP categories between two groups of individuals (Caucasian and Taiwanese) using probe ratio data for a multi-allelic CNP probe in the *FCGR3* gene on Chromosome 1. We also apply the chi-square test of independence to the probe ratio data after the individuals are classified into categories using the Bayesian classification rule described above. Oligonucleotide probes are designed as described previously [43].

To be consistent with notation used throughout this work, from this point forward we label the Taiwanese samples as “controls” and the Caucasian samples as “cases”, although individuals in this study were not ascertained for any particular disease phenotype.

## Results

### Simulation studies to verify asymptotic null distribution of chi-square test with Bayesian classification

In Table 1, we report the *empirical type I error rates* at the 0.975, 0.10, 0.05, 0.025, and 0.01 significance levels for each set of parameter settings. For each simulation, these type I error rates are the proportion of replicates for which the computed *LRTS* exceeds 0.2157, 6.25, 7.81, 9.348 or 11.34, which correspond to the 0.975, 0.10, 0.05, 0.025 and 0.01 significance level cutoffs for a central chi-square distribution with 3 degrees of freedom (the asymptotic null distribution for each simulation). For each empirical type I error rate, we report the 95% confidence interval, based on 1000 replicates. As an additional confirmation, we apply the Kolmogorov-Smirnoff (KS) goodness of fit test [44], [45] to each simulations' set of 1000 *LRTS* values (i.e., sample size for KS test is 1000), and report the p-values in Table 1.

**Table 1:** Simulation results of the null distribution of chi-squared test.

Sample size	Proportions	Empirical type I error rate*					KS-Test P-value
		0.975 Level	0.10 Level	0.05 Level	0.025 Level	0.01 Level	
200	(0.25, 0.25, 0.25, 0.25)	0.976	0.107	0.042	0.018	0.005	0.72
500	(0.25, 0.25, 0.25, 0.25)	0.971	0.092	0.047	0.025	0.007	0.78
200	(0.1, 0.2, 0.3, 0.4)	0.979	0.094	0.046	0.018	0.006	0.54
500	(0.1, 0.2, 0.3, 0.4)	0.983	0.106	0.056	0.036	0.010	0.36

Based on 1000 replications for each settings.

In each simulation, the target type I error rate is contained in the 95% confidence interval for the corresponding empirical type I error rate. In addition, the smallest KS test p-value is 0.36, indicating that we do not reject the null hypothesis that the data are drawn from a central chi-square distribution with 3 degrees of freedom.

### Computing asymptotic power for LRTS of copy number measurement

When the alternative hypothesis 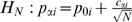 is true, the NCP of the *LRTS* may be written in a quadratic form as:
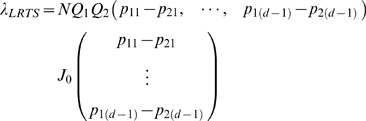
where *J*_0_ is the (*d*−1)×(*d*−1) symmetric matrix specified in Appendix S1.

### Simulation studies to verify asymptotic null and alternative distributions of LRTS

As in Table 1, in Table 2, we report the empirical type I error rates at the 0.10, 0.05, and 0.01 significance levels for each set of parameter settings. For each simulation, these type I error rates are the proportion of replicates for which the computed *LRTS* exceeds 6.25, 7.81, or 11.34, which correspond to the 0.10, 0.05, and 0.01 significance level cutoffs for a central chi-square distribution with 3 degrees of freedom (the asymptotic null distribution for each simulation). For each empirical type I error rate, we report the 95% confidence interval, based on 1000 replicates. As an additional confirmation, we apply the Kolmogorov-Smirnoff (KS) goodness of fit test [44], [45] to each simulations' set of 1000 *LRTS* values (i.e., sample size for KS test is 1000), and report the p-values in Table 2.

**Table 2:** Simulation results of the null distribution of *LRTS*.

Sample size	Proportions	Empirical type I error rate*					KS-Test P-value
		0.975 Level	0.10 Level	0.05 Level	0.025 Level	0.01 Level	
200	(0.25, 0.25, 0.25, 0.25)	0.979	0.103	0.045	0.015	0.007	0.81
500	(0.25, 0.25, 0.25, 0.25)	0.971	0.097	0.052	0.021	0.013	0.79
200	(0.1, 0.2, 0.3, 0.4)	0.977	0.106	0.046	0.020	0.005	0.34
500	(0.1, 0.2, 0.3, 0.4)	0.982	0.109	0.060	0.028	0.011	0.41

Based on 1000 replications for each settings.

In each simulation, the target type I error rate is contained in the 95% confidence interval for the corresponding empirical type I error rate. In addition, the smallest KS test p-value is 0.34, indicating that we do not reject the null hypothesis that the data are drawn from a central chi-square distribution with 3 degrees of freedom.

In Table 3, we report the *simulation power* at the 10^−3^, 10^−4^, and 10^−5^ significance levels for each set of parameter settings. For each simulation, these powers are the proportion of replicates for which the computed *LRTS* exceeds 16.27, 21.11, or 25.90, which correspond to the 10^−3^, 10^−4^, and 10^−5^ cutoffs for a central chi-square distribution with 3 degrees of freedom (the asymptotic null distribution for each simulation). More stringent significance level cutoffs are chosen for the power analyses since power at the 0.10, 0.05, and 0.01 levels is close to or equal to 100% for these parameter specifications. As with the empirical type I error rates in Table 1, we report the 95% confidence intervals, based on 1000 replicates each. We also report the asymptotic power at each of the significance levels, determined by computing the non-centrality parameter (equation (A1)) for each set of parameter settings. As an additional confirmation, we apply the Kolmogorov-Smirnoff (KS) goodness of fit test [44], [45] to each simulations' set of 200 *LRTS* values (i.e., sample size for KS test is 200), and report the p-values in Table 3.

**Table 3:** Simulation results for *LRTS* under alternative distributions.

MOI	Method to calculate power	*Simulation Power* *			KS-Test P-value
		10^−3^ Level	10^−4^ Level	10^−5^ Level	
Dosage	Simulation	0.958 (0.946, 0.970)	0.866 (0.845, 0887)	0.735 (0.708, 0.762)	0.01
	Asymptotic	0.949	0.856	0.712	
Extremes	Simulation	0.950 (0.936, 0.964)	0.857 (0.835, 0.879)	0.738 (0.711, 0.765)	0.07
	Asymptotic	0.946	0.848	0.700	

Legend for Table 2. Based on 1000 replications and 200 sample size per case/control group.

* 95% approximate confidence intervals for simulated power are given in parentheses.

Here, we present simulated and asymptotic power for the *LRTS* when the alternative hypothesis that mixing proportions are different in each of two groups is true. The mixing proportions are computed using equations (4) for the Dosage and Extremes models, where CNP population frequencies are as specified above (Methods - Genetic model parameters for efficiency analysis). For the Dosage model, the relative risks are: *R*_2_ = 1.8, *R*_3_ = 1.8^2^ = 3.64, *R*_4_ = 1.8^3^ = 5.83. For the Extremes model, the relative risks are: *R*_1_ = 1, *R*_2_ = 0.3, *R*_3_ = 0.3, *R*_4_ = 1. Asymptotic power is computed using the non-centrality parameter documented in equation (A1). The column “KS-Test P-value” refers to the p-value computed using the Kolmogoroff-Smirnoff goodness of fit test, as implemented in R programming environment.

While the KS p-values are much smaller, we see that, for the 10^−3^ and 10^−4^ significance levels, the simulation power is contained in the 95% confidence interval for each simulation. The results of this table suggest that our simulation results are consistent with asymptotic results for at least the 10^−3^ and 10^−4^ significance levels.

### Relative efficiency of the 2×*d* chi-square test relative to the LRTS

**Figure 2:**
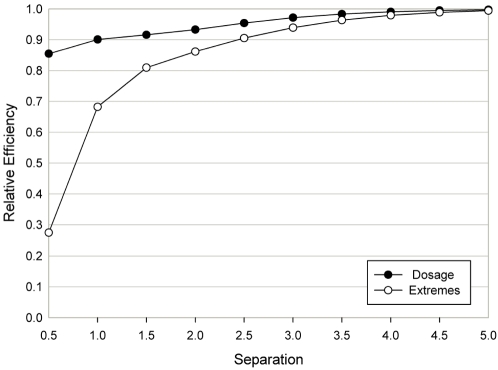
Here we present the relative efficiency *Eff* (defined in Methods) of the chi-square test of independence in relation to the *LRTS* as a function of separation (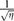) between the four component distributions that comprise the mixture distribution. All information regarding parameter specification for the Dosage and Extremes models for which relative efficiencies are calculated is presented in the Methods section (Genetic model parameters for efficiency analysis).

Using the result for the NCP of the *LRTS*,
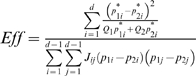
, where 
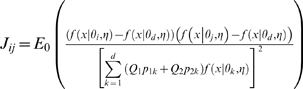
. Figure 2 contains the relative efficiency of the 2×4 chi-square test with Bayesian rule classification with respect to the *LRTS* for the Extremes and Dosage models against the separation between successive category means. In all models, the relative efficiency is less than 1; that is, the *LRTS* is more powerful. When the separation is 5 standard deviations or greater, both tests have essentially the same power. The relative efficiency steadily declines as the separation between category means decreases, with less efficiency for the Extremes model.

### Example CNP data for two populations

**Figure 3:**
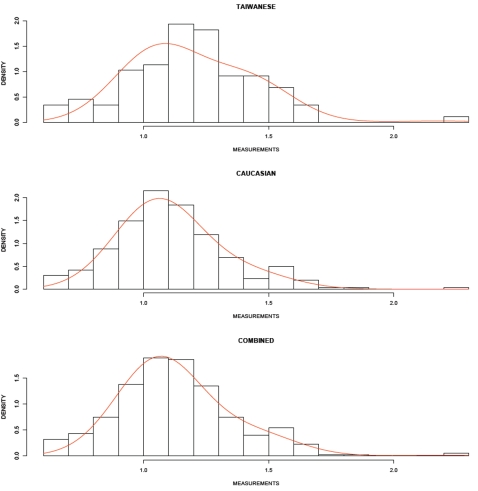
In these figures, we provide histograms of P4077 probe ratio data for Taiwanese, Caucasian and Combined (Taiwanese and Caucasian) samples. We also provide a fitted probability density function line for each data set. These graphs were created using the R programming environment. The horizontal axis labeled “MEASUREMENT” refers to each individual's probe ratio data value (after log transform) for the P4077 probe.

Results for the *LRTS* applied to P4077 probe ratio data for the Caucasian and Taiwanese samples are presented in Table 4. Figure 3 contains the histograms of each group's probe ratio data, as well as of the combined groups (Caucasians and Taiwanese). There are an estimated three CNP categories, and the *LRTS* p-value for the P4077 probe is 0.014. In comparison, the chi-square test of independence p-value based on the asymptotic null distribution for the P4077 probe data with classification by the Bayesian rule is 0.03. The p-value based on Fisher's Exact Test is 0.0175. The numbers of Caucasian and Taiwanese individuals in CNP categories 1, 2, and 3 are: 229, 31, and 1; and 67, 20, and 1, respectively, as determined by the Bayesian classification rule. Additionally, we report the estimated classification rates as follows:
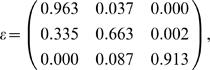
where *ε_ij_* = Pr(*reported CNP classification* = *j*|*true CNP classification* = *i*). The *LRTS* method provides a slightly more significant p-value.

**Table 4:** Parameter estimation with 3 component normal mixtures for probe P4077 ratio data.

Hypothesis	Estimated parameters	CNP Category		
		*i* = 1	*i* = 2	*i* = 3
Null (*H*_0_)	Mixing proportions	0.815	0.179	0.006
	Means (*θ_i_*)	1.062	1.446	2.191
Alternative (*H_N_*)	Mixing proportions for Taiwanese (*p*_1*i*_)	0.626	0.362	0.011
	Mixing proportions for Caucasians (*p*_2*i*_)	0.843	0.152	0.005
	Means (*θ_i_*)	1.056	1.420	2.180

Legend for Table 4. Data are determined for 261 individuals of Caucasian ethnicity and 88 individuals of Taiwanese ethnicity. The estimated variance (*η*) under both the null and alternative hypotheses is 0.03.

When we use the estimated misclassification parameters in the matrix *ε* along with the estimated mixing proportions under the alternative hypothesis (Table 4) in the *P*ower for *A*ssociation *W*ith *E*rror (PAWE) webtool, the power at the 5% significance level for the sample sizes specified in our example is 98% with error-free data, and is 76% with error rates given in *ε*, a power loss of 22%. From the perspective of power loss, Kang et al. [30], [31] showed that misclassification of the most common category to any other category is the most costly; here, the estimated error rate of 3.7% in classification CNP category “1” as category “2” results in the greatest power loss. Other investigators have previously documented that the chi-square test of independence and the linear trend test lose power under such misclassification when data are genotypes or multi-locus haplotypes [30], [31], [46], [47].

Additionally, if we compute the separation values 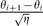, *i* = 1, 2, using the estimated parameters from Table 3, we see that separation between categories 1 and 2 is 
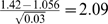
, and separation between categories 2 and 3 is 
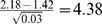
. That is, for the majority of samples (categories 1 and 2) the separation is only 2.09. Our results of the relative efficiency studies in Figure 2 also suggest that, for such separation, the chi-square test with Bayesian classification is a less powerful procedure than the *LRTS*.

While one cannot use parameters estimated from data collected to calculate actual power, we present these calculations as indications of the source of the greater power of the *LRTS* due to the relatively high misclassification rates that are consistent with the estimated parameters.

## Discussion

We have derived the non-centrality parameter for the *LRTS* of the mixture proportions applied to the CNP quantitative measurements. The relative efficiency of the 2×4 chi-square test is less than 1 for the example disease MOIs considered here, with greater decreases as the separation between category-means decreases. That is, for the models considered, the *LRTS* is more powerful than the chi-square test. In the example, power may have been lost for the chi-square test because of relatively high estimated misclassification rate from the most common category to the second most common category. The chi-square test of independence can lose substantial power under such misclassification [30], [31], [47].

A key advantage of the *LRTS* is that it can be computed on *any* CNP data, whether or not that data can be categorized. While the example presented (Table 4 and Figure 3) used only a single CNP as an illustration, the *LRTS* can be calculated for multiple SNPs analyzed simultaneously through specification of a multivariate pdf. The formal statistical analysis is the same, in that the *LRTS* is calculated as shown in Equation (3). Additionally, extensions of a multivariate procedure can incorporate more complex modeling of the mixture mechanism, for example, including a Hidden Markov Model approach.

The results indicated in Table 1 and Figure 2, namely that non-differential misclassification errors do not result in a change in the type I error rate and that there is power loss for the chi-square test of association, are consistent with numerous publications on the subject of non-differential genotyping error. Pompanon et al. [48] and Gordon and Finch [49], [50] provide reviews of the literature.

As an alternative analysis, one might consider a logistic regression model with case/control status as the dependent variable and CNP quantitative measure as the independent variable. One potential advantage of this method is that determination of optimal estimates is less computationally intensive than the LRTS procedure documented in this work. Another potential advantage of logistic regression is that it allows for the possible inclusion of covariates. In this work we focus on the *LRTS* to avoid specification of a mathematical model of association. That is, the *LRTS* presented here only tests whether mixing proportions are different in two groups. There are mixture models that examine whether covariates are associated with CNP category membership [51], [52]. A natural next step to extend our work is to allow the inclusion of covariates. The *LRTS* is similar in spirit to the commonly used chi-square test of independence for genotype data on cases and controls. That statistic similarly tests for differences in allele or genotype frequencies among different categories (e.g., cases and controls). We further note that there is literature on power and sample size for logistic regression [53], [54]. While robustness of logistic regression procedures when the independent variable is drawn from a single univariate normal distribution is well documented (e.g., see [55]), the extension to logistic regression procedures when the independent variable is drawn from a mixture of distributions, as is the situation with CNPs, needs further investigation.

The recent work documenting differences in CNP distributions for different ethnic populations is consistent with the frequently replicated results that there are different allele and genotype frequency distributions in different ethnic populations [13], [56]. Yu et al. [57] confirmed CNP values with “gold-standard” sequencing data. It is a limitation of our example that our estimated CNP classifications are not confirmed with sequencing data. Recent methodological research has documented several benefits of having standard and gold-standard measurements simultaneously on a subset of individuals [58], [59], [60]. Such sampling has been referred to as double-sampling [61], [62].

An additional limitation in the data analysis of our example is our assumption of equal variances among the component distributions. While this assumption appeared to be true for this example, it will not hold in general. In that event, methods such as those proposed by Hathaway [33], [34] may be used.

The power and sample size calculations presented here are based on asymptotic theory; that is, our results should hold when sample sizes are sufficiently large. When sample sizes are smaller, one can use simulation methods to estimate power. Of course, p-values should be based on permutation tests in such instances.

### Web Resources

Online Mendelian Inheritance in Man (http://www.ncbi.nlm.nih.gov/Omim)

Power for Association With Error (http://linkage.rockefeller.edu/pawe/)

## Supporting Information

Appendix S1 (0.02 MB PDF)
