# Supplementary material for: Computing Power and Sample Size for Case-Control Association Studies with Copy Number Polymorphism: Application of Mixture-Based Likelihood Ratio Test
Source: PLoS One. 2008 Oct 22;3(10):e3475. doi: 10.1371/journal.pone.0003475 (PMC2566806; doi:10.1371/journal.pone.0003475)
Supplement: Appendix S1 — (0.02 MB PDF) [file pone.0003475.s001.pdf]

Appendix S1

To derive the NCP of the *LRTS*, let $\vec{p}_0 = (p_{01}, \cdots, p_{0d})$ denote the true parameter under H_0. Since $\sum_{i=1}^{d} p_{\alpha i} = 1$ for $\alpha = 1$ or 2, we use $p_{\alpha d} = 1 - \sum_{i=1}^{d-1} p_{\alpha i}$. The notation I_0 denotes the Fisher information matrix [37] under H_0. It can be written as

$I_0 = \begin{pmatrix} K_0 & A_0 \\ A_0^T & B_0 \end{pmatrix}$ where B_0 is symmetric, $K_0 = \begin{pmatrix} J_0 & 0 \\ 0 & J_0 \end{pmatrix}$, and

$$J_0 = E_0 \begin{pmatrix} \left(\frac{f(x|\theta_1,\eta) - f(x|\theta_d,\eta)}{\sum p_{0i} f(x|\theta_i,\eta)} \right)^2 & \cdots & \frac{[f(x|\theta_1,\eta) - f(x|\theta_d,\eta)][f(x|\theta_{d-1},\eta) - f(x|\theta_d,\eta)]}{\left(\sum p_{0i} f(x|\theta_i,\eta)\right)^2} \\ \vdots & \ddots & \vdots \\ \frac{[f(x|\theta_1,\eta) - f(x|\theta_d,\eta)][f(x|\theta_{d-1},\eta) - f(x|\theta_d,\eta)]}{\left(\sum p_{0i} f(x|\theta_i,\eta)\right)^2} & \cdots & \left(\frac{f(x|\theta_{d-1},\eta) - f(x|\theta_d,\eta)}{\sum p_{0i} f(x|\theta_i,\eta)} \right)^2 \end{pmatrix}.$$

Consider a family of hypotheses $H_N : p_{\alpha i} = p_{0i} + \frac{c_{\alpha i}}{\sqrt{N}}$. By decomposing a vector $v = (c_{11}\sqrt{Q_1}, \cdots, c_{1(d-1)}\sqrt{Q_1}, c_{21}\sqrt{Q_2}, \cdots, c_{2(d-1)}\sqrt{Q_2})^T$ into a sum of two orthogonal vectors $v^{\parallel}$ and $v^{\perp}$ with respect to the inner product defined by $\langle a,b \rangle = a^T K_0 b$ for vectors a and b. The vector $v^{\parallel}$ is the orthogonal projection of v onto the space of the null hypotheses.

$$v = \begin{pmatrix} \frac{c_{11}Q_1 + c_{21}Q_2}{Q_1 + Q_2}\sqrt{Q_1} \\ \vdots \\ \frac{c_{1(d-1)}Q_1 + c_{2(d-1)}Q_2}{Q_1 + Q_2}\sqrt{Q_1} \\ \frac{c_{11}Q_1 + c_{21}Q_2}{Q_1 + Q_2}\sqrt{Q_2} \\ \vdots \\ \frac{c_{1(d-1)}Q_1 + c_{2(d-1)}Q_2}{Q_1 + Q_2}\sqrt{Q_2} \end{pmatrix} + \begin{pmatrix} \frac{(c_{11} - c_{21})\sqrt{Q_1Q_2}}{Q_1 + Q_2}\sqrt{Q_2} \\ \vdots \\ \frac{(c_{1(d-1)} - c_{2(d-1)})\sqrt{Q_1Q_2}}{Q_1 + Q_2}\sqrt{Q_2} \\ -\frac{(c_{11} - c_{21})\sqrt{Q_1Q_2}}{Q_1 + Q_2}\sqrt{Q_1} \\ \vdots \\ -\frac{(c_{1(d-1)} - c_{2(d-1)})\sqrt{Q_1Q_2}}{Q_1 + Q_2}\sqrt{Q_1} \end{pmatrix}$$
$$= v^{\parallel} + v^{\perp}.$$

Since $(v^{\parallel})^T K_0 v^{\perp} = 0$ and $v = v^{\parallel} + v^{\perp}$, the NCP is the quadratic form $(v^{\perp})^T K_0 v^{\perp}$:

$$\lambda_{LRTS} = Q_1 Q_2 \sum_{i=1}^{d-1} \sum_{j=1}^{d-1} J_{ij} (c_{1i} - c_{2i})(c_{1j} - c_{2j}) \,.$$

Using the relationship $c_{\alpha i} = \sqrt{N}(p_{\alpha i} - p_{0i})$,

$$\lambda_{LRTS} = N Q_1 Q_2 \sum_{i=1}^{d-1} \sum_{j=1}^{d-1} J_{ij} (p_{1i} - p_{2i})(p_{1j} - p_{2j}) \,. \qquad \text{(A1)}$$

This completes the derivation of the NCP of the *LRTS*.
